# Supplementary material for: Remimazolam versus propofol for procedural sedation: a meta-analysis of randomized controlled trials
Source: PeerJ. 2023 Jun 12;11:e15495. doi: 10.7717/peerj.15495 (PMC10269568; doi:10.7717/peerj.15495)
Supplement: Supplemental Information 2 [file peerj-11-15495-s002.docx]

**Supplementary Materials**

**Supplemental Figure 1.** Forest plot demonstrates the odds ratio of bradycardia between the remimazolam group and the propofol group.


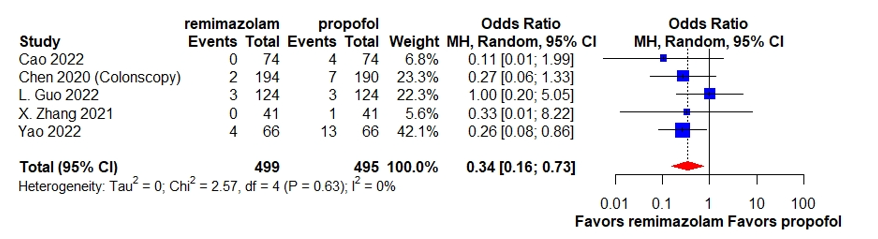


**Supplemental Figure 2.** Forest plot demonstrates the odds ratio of hypotension between the remimazolam group and the propofol group.


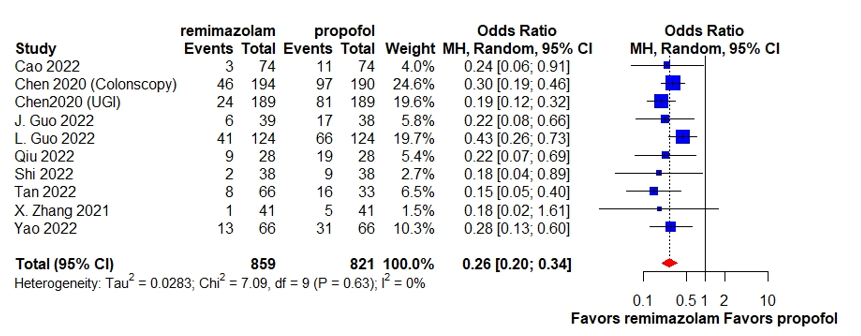


**Supplemental Figure 3.** Forest plot demonstrates the odds ratio of respiratory depression between the remimazolam group and the propofol group.


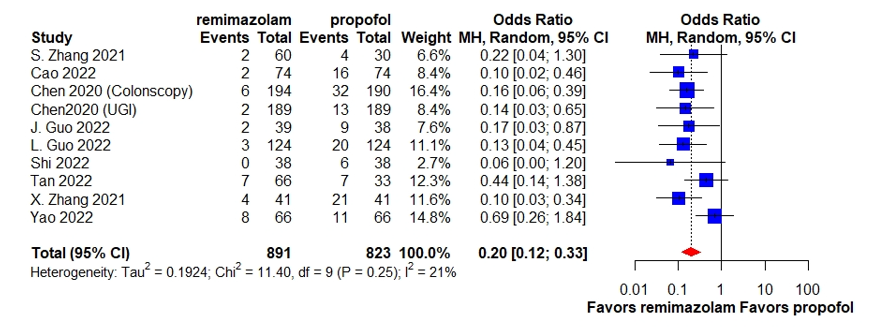


**Supplemental Figure 4.** Forest plot demonstrates the odds ratio of postoperative nausea and vomiting between the remimazolam group and the propofol group.


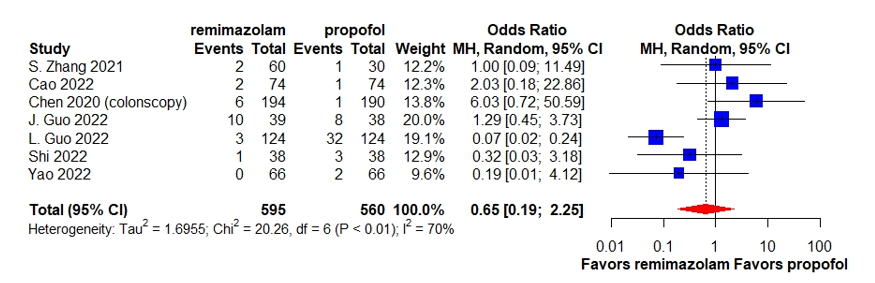


**Supplemental Figure 5.** Forest plot demonstrates the odds ratio of dizziness between the remimazolam group and the propofol group.


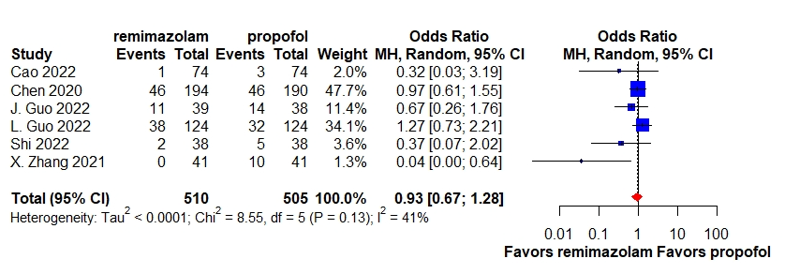


**Supplemental Figure 6.** Forest plot demonstrates the odds ratio of injection pain between the remimazolam group and the propofol group.


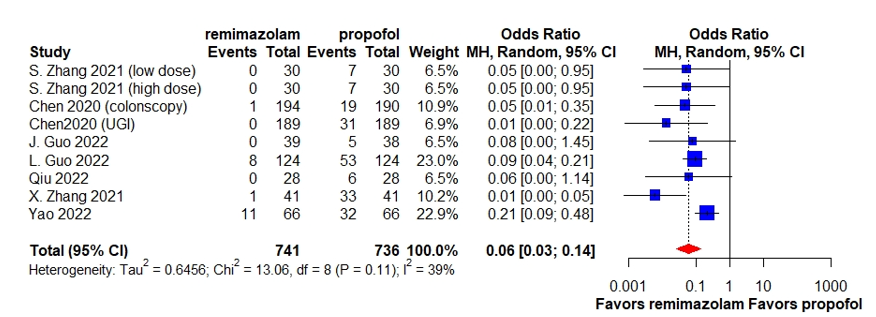


**Supplemental Figure 7.** Forest plot demonstrates the odds ratio of sedation success rate between the remimazolam group and the propofol group.


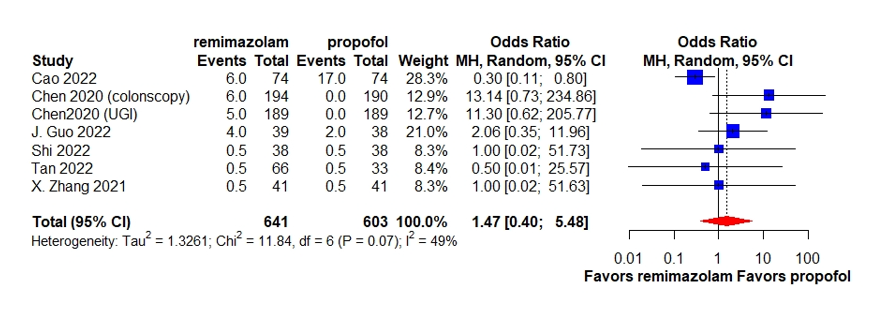


**Supplemental Figure 8.** Forest plot demonstrates the difference in time to loss of consciousness between the remimazolam group and the propofol group.


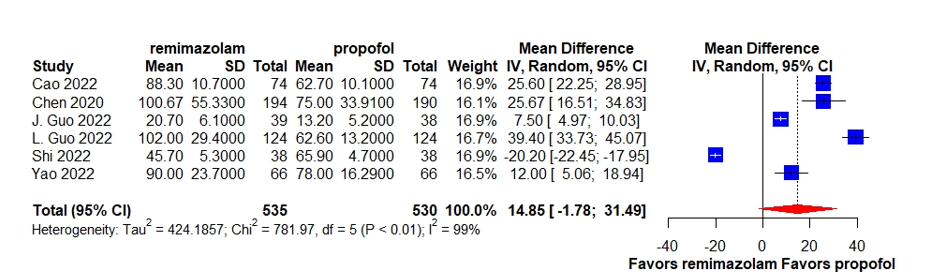


**Supplemental Figure 9.** Forest plot demonstrates the difference in time to recovery between the remimazolam group and the propofol group.


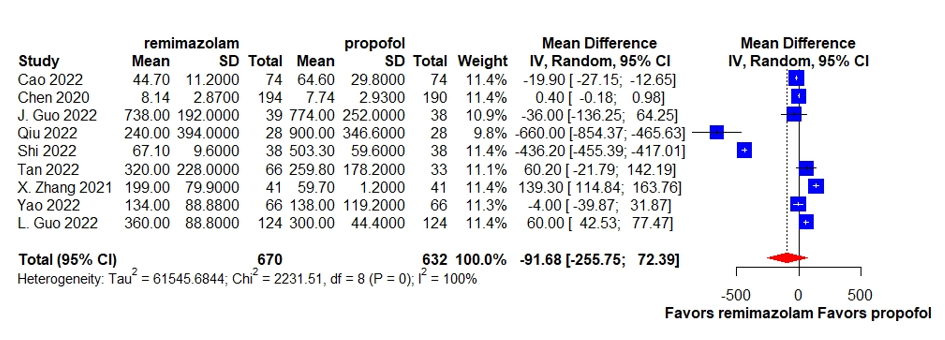


**Supplemental Figure 10.** Forest plot demonstrates the difference in time to discharge between the remimazolam group and the propofol group.

**Supplemental method1.** Search strategy

***Embase***

| Search Number | Search Description | Numbers of results |
| --- | --- | --- |
| 1 | (procedur* or laryngoscopy or bronchoscopy or bone marrow aspiration or biopsy or catheterization or gastroscopy or panendoscopy or colonoscopy or sigmoidoscopy or endoscop* or ERCP or ERBD or EUS or cystoscopy or hysteroscope or uteroscope or colposcope):ti,ab,kw,de | 1709347 |
| 2 | 'medical procedures '/exp | 28039146 |
| 3 | 'procedural sedation '/exp | 46 |
| 4 | 'remimazolam'/exp | 278 |
| 5 | (remimazolam):ti,ab,kw,de | 290 |
| 6 | 'Propofol'/exp | 65022 |
| 7 | (Propofol):ti,ab,kw,de | 67471 |
| 8 | (#1 OR #2 OR #3 ) AND (#4 OR #5) AND (#6 OR #7)and [embase]/lim | 131 |

***Medline***

| Search Number | Search Description | Numbers of results |
| --- | --- | --- |
| 1 | (procedur* or laryngoscopy or bronchoscopy or bone marrow aspiration or biopsy or catheterization or gastroscopy or panendoscopy or colonoscopy or sigmoidoscopy or endoscop* or ERCP or ERBD or EUS or cystoscopy or hysteroscope or uteroscope or colposcope).mp | 2478173 |
| 2 | (remimazolam).mp | 229 |
| 3 | exp "propofol"/ | 16272 |
| 4 | (Propofol).mp | 24896 |
| 5 | 1 AND 2 AND (3 OR 4) | 131 |
| [mp=title, abstract, original title, name of substance word, subject heading word, floating sub-heading word, keyword heading word, organism supplementary concept word, protocol supplementary concept word, rare disease supplementary concept word, unique identifier, synonyms] | | |

***Cochrane***

***
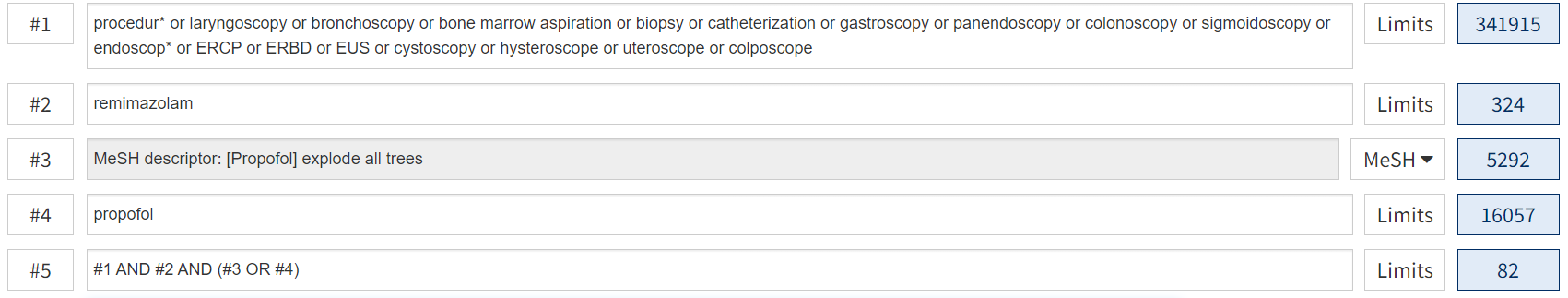
***

**Supplemental method2.** Data synthesis

We used Rstudio with metafor packages to conduct statistical analysis**【metafor】package**
**Random-effects model**

**Restricted maximum likelihood (REML) method as a heterogeneity estimator**

**Several plots for meta-analysis: Forest plot (forest)**

***Binary outcomes***

metaresult<-metabin(event.e, n.e, event.c ,n.c, studlab=Study,sm="OR",data=data,method="MH",method.tau="REML",comb.random=TRUE,comb.fixed=FALSE)

***Continuous outcomes***

metaresult<-metacont(n.e, mean.e, sd.e,n.c, mean.c, sd.c,studlab=year,sm="MD",data=data,method.tau="REML",comb.random=TRUE,comb.fixed=FALSE)

***Forest plot***

forest (metaresult, layout="RevMan5", lab.e="remimazolam ", lab.c="propofol", xlab="Favors remimazolam Favors propofol ", ff.xlab="bold", col.by="black", pooled.events=F, comb.random=T, comb.fixed=F, col.diamond.random=("red"), col.diamond.lines.random="red", col.square = 'blue', col.square.lines = 'blue')
