# Supplementary material for: Remimazolam versus propofol for procedural sedation: a meta-analysis of randomized controlled trials
Source: PeerJ. 2023 Jun 12;11:e15495. doi: 10.7717/peerj.15495 (PMC10269568; doi:10.7717/peerj.15495)
Supplement: Supplemental Information 3 [file peerj-11-15495-s003.docx]

**Systematic Review andor Meta-Analysis Rationale**

With a completely different mechanism compared to propofol, remimazolamis a new short-acting GABA-A receptor agonist. It is an ester-based benzodiazepine (BZD) and can be rapidly hydrolyzed into inactive metabolites by tissue esterases. The onset of remimazolam is approximately one to three minutes and with short metabolic half-life as well, thereby providing adequate moderate sedation but faster recovery after intervention. There was a recent published meta-analysis compared safety and efficacy of remimazolam versus propofol^1^, however, the authors included not only patients undergoing procedural sedation, but also general surgery and heart surgery. There was obvious conceptual heterogeneity caused by great variation of the included studies in the aforementioned review by Zhang et al. Moreover, there are emerging studies reporting remimazolam use for procedural sedation compared to propofol. Therefore, we aim to conduct a systematic review and meta-analysis to update and clarify the efficacy and safety of remimazolam versus propofol for procedure sedation.

Reference:

1. Zhang J, Cairen Z, Shi L, et al. Remimazolam versus propofol for procedural sedation and anesthesia: a systemic review and meta-analysis. Minerva Anestesiol. 2022;88(12):1035-1042.
